# Effects of Microencapsulated Essential Oils on Equine Health: Nutrition, Metabolism and Methane Emission

**DOI:** 10.3390/life13020455

**Published:** 2023-02-06

**Authors:** Mona M. M. Y. Elghandour, Aristide Maggiolino, Erendira Itzel Ceja García, Pedro Sánchez-Aparicio, Pasquale De Palo, José Luis Ponce-Covarrubias, Alberto Barbabosa Pliego, Abdelfattah Z. M. Salem

**Affiliations:** 1 Facultad de Medicina Veterinaria y Zootecnia, Universidad Autónoma del Estado de México, Toluca 50000, Estado de México, Mexico; 2 Department of Veterinary Medicine, University of Bari Aldo Moro, 70010 Valenzano, Italy; 3 Facultad de Ciencias, Universidad Autónoma del Estado de México, Toluca 50000, Estado de México, Mexico; 4 Escuela Superior de Medicina Veterinaria y Zootecnia No. 3, Universidad Autónoma de Guerrero (UAGro), Técpan de Galeana 40900, Guerrero, Mexico

**Keywords:** equine, essential oils, microencapsulated, metabolism, methane

## Abstract

This review examines the available data regarding the positive effects of microencapsulated essential oils (EOs) on the nutrition, metabolism, and possibly the methane emission of horses. A literature review was conducted on the effect of microencapsulated (EOs) on the health of horses. The information comprises articles published in recent years in indexed journals. The results indicate that mixtures of microencapsulated EOs may be beneficial to equine health due to their antimicrobial and antioxidant activity, as well as their effects on enteric methane production, nutrient absorption, and immune system enhancement. Moreover, encapsulation stabilizes substances such as EOs in small doses, primarily by combining them with other ingredients.

## 1. Introduction

Essential oils (EOs) are natural volatile complexes of aromatic plants. The most effective method for removing these essences is to drag them through vaporized water extracted from aromatic plants [1,2]. EOs are complex mixtures of volatile compounds, composed primarily of aliphatic molecules that form esters, aldehydes, alcohols, alkanes, and ketones. In addition to sesquiterpenes, phenylpropanoids and monoterpenes [3,4,5,6] are the compounds most commonly used in pharmaceutical, agricultural, and cosmetics industries.

Maintaining good health is one of the benefits of EOs when they are included in the diet due to their action in the digestive system, where they enhance the absorption of nutrients. EOs have been used for centuries in traditional folk medicine for humans and animals for nutritional management [3]. EOs have been inhaled as vapors or rubbed onto the skin, resulting in positive health effects [7]. The topical application of EOs produces antibacterial, anti-inflammatory, and analgesic effects [7,8]. EOs have a sedative and stimulating effect on animals, as well as a positive effect on their behavior and immune system. Due to their modulating effect on intestinal microbiota, the use of EOs in animal feeding to improve productive performance, health, and nutrient absorption has become fairly common [9,10,11]. However, the mechanisms underlying these functions in horse diets have not yet been fully elucidated. In ruminant feed, essential oils have a lot of available compounds, are novel feed additives, and have effects on ruminal fermentation and metabolism [12]. Castillejos, Calsamiglia, Martín-Tereso, and Ter Wijlen [12] evaluated a series of thymol doses on rumen microbial fermentation and found that 500 and 5000 mg/L thymol decreased ammonia concentration due to the inhibition of ruminal microorganisms’ activities. Therefore, the enrichment of horse diets with EOs and their processing—constituting a valuable perspective for enhancing equine performance, including digestion, metabolism, and their enteric emission into the environment—can be considered beneficial.

Microencapsulation is the most efficient method used in the processing of EOs. Microencapsulation is defined as the method of packaging in which small particles of liquid or gas are encapsulated by simple blend operations or complex polymeric coating systems. The result is the formation of microcapsules with a semipermeable, thin, and resilient membrane. The substances of interest, EOs, can be covered and contained by polymeric materials. Similar to microencapsulating alginate-based polymers, microencapsulating EOs may require maintaining the viability of the encapsulated compounds and preserving them in a large number of EO mixtures [13,14]. The primary objective of this review is to look into the impact of microencapsulated EOs on methane emission in horses, as well as their positive effects on equine health, primarily by manipulating the microbiota of the gastrointestinal tract.

## 2. Enteric Fermentation in Horses

**Figure 1:**
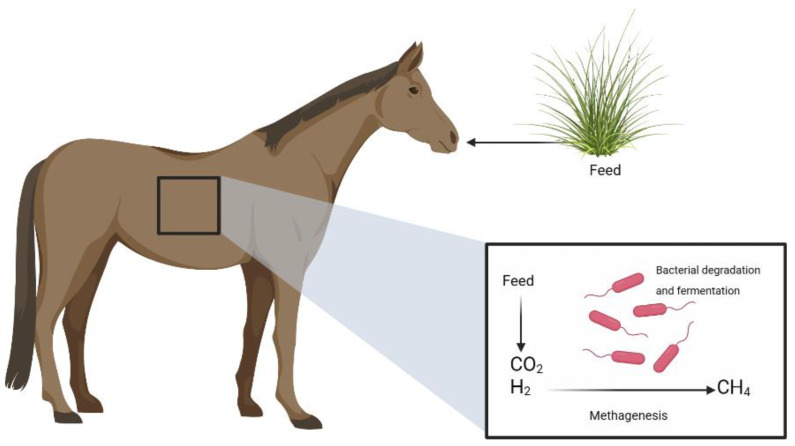
Diagram of the enteric fermentation process in horses.

This process is carried out in the digestive system by microorganisms that degrade biomolecules via anaerobic fermentation. Enteric fermentation is the largest contributor to livestock emissions. CH_4_ is one of the products that can be exhaled or expelled from the terminal end of the digestive tract. Other products include acetic acid and carbon dioxide. Equines are cecum–colon fermenting animals that produce CH_4_. Consequently, methanogenesis occurs in the cecum and colon. Here, cellulolytic, and methanogenic bacteria are abundant, and they proliferate in the presence of a cellulose-rich diet that is easily digested, resulting in an increase in enteric CH_4_ fermentation [10,15]. Several factors increase or decrease the emission of enteric CH_4_; these include the low digestibility of feed, which causes an increase in enteric CH_4_ emission, the frequency of daily feed intake, and the lower presence of enteric bacteria. Other factors that affect CH_4_ emissions are the swallowing rate and the amount of food consumed, which are dependent on the animal’s size and activity level [16]. Various factors, including diet, influence CH_4_ production in horses, feed digestibility, pre-processing of feed, and the frequency with which they consume it; therefore, nutritional manipulation could be an option for the mitigation or suppression of methanogenesis. Essential and probiotic oils are among the solutions to reduce CH_4_ production; this practice would likely reduce CH_4_ emissions because it could modify ruminal fermentation by directly inhibiting the methanogenic microorganisms responsible for it and by diverting hydrogen ions away from methanogens [17] (Figure 1).

Due to their biochemical activity, enteric bacteria produce a large number of substances; many of these substances are toxic, and equines must perform detoxification to neutralize them, incurring an energy expenditure in the process. Nonetheless, it is closely associated with the quality of the feed and antimicrobial or probiotic additives, as well as the horse’s cleanliness and hygiene [18].

## 3. Essential Oils (EOs)

The EOs are a mixture of small volatile compounds, typically liquid and colorless, obtained as a by-product of plants and seeds from flowers, barks, petals, fruits, stems, leaves, and roots. There are approximately 3000 distinct kinds of EOs, and the quantity of oils that plants produce depends on optimal environmental conditions and the stage of plant development [10].

**Figure 2:**
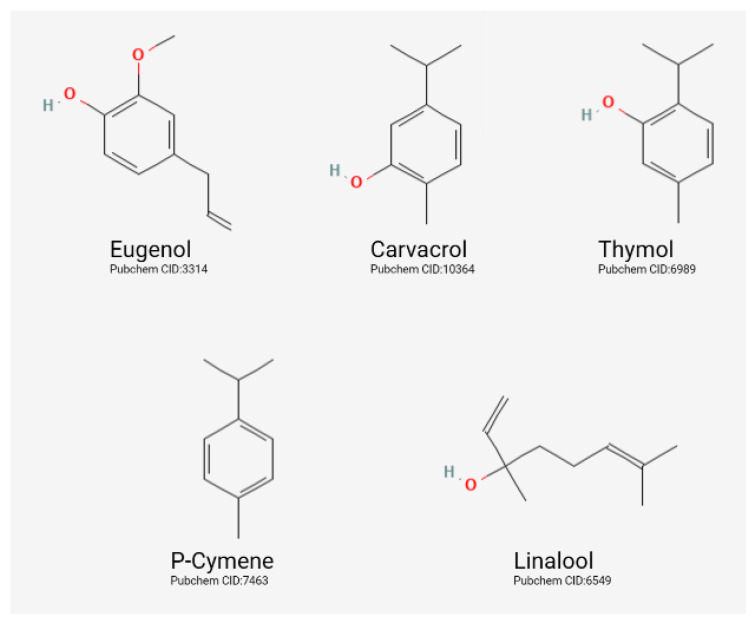
Representation of the chemical structures of some of the most common essential oil compounds.

The aroma exuded by plants is essential to the process of pollination. In EOs, various compounds attract pollinating insects to their host; among these compounds are hydrocarbons (terpenes), as well as oxygenated compounds such as alcohols, esters, aldehydes, and phenolic compounds, owing to their ability to impart aroma to EOs. Compounds in mixtures can range from two to one hundred, with the majority being alkanes, aldehydes, alcohols, esters, acids, ketones, monoterpenes, phenylpropane, and sesquiterpenes. In addition, some of these EOs’ structures (Figure 2) contain thymol, Y-terpinene, carvacrol, sabinene, -thujene, p-cymene, -terpinene, eugenol, and linalool [13,19]. The EOs can have various origins (synthetic, natural, artificial). Natural products are direct plant products, so distillation with water vapor or volatile solvents does not alter their chemical or physical structure [19].

Some EOs have pharmacological effects, including antioxidant, anti-inflammatory, and anti-cancer properties, as can be seen in Table 1 [12,13]; other EOs have antimicrobial properties against protozoa, bacteria, and viruses [14], and some EOs even kill insects [16]. EOs have a multifaceted effect on the microbiological environment. It depends primarily on their hydrophilic or lipophilic properties. Enzyme-catalyzed membrane activities are influenced by terpenoids, a type of fat-soluble agent. In this instance, their action can be seen taking place in the airways. Components of EOs that can act as decouplers can disrupt the phosphorylation of ADP (primary energy of metabolism) by interfering with the translocation of protons across membrane vesicles [20,21].

Considering their molecular structure, the extremely complex mixture of volatile molecules that EOs generate can be divided into two main categories. The first group consists of phenylpropanoids, while the second consists primarily of terpenoids and terpenes. Terpenoids and terpenes are hydrocarbons formed by combining varying amounts of isoprene. Although many terpenes lack antimicrobial activity, some possess potent micromynides [36,37]. Thymol, methanol, linalool, carvacrol, linalyl acetate, geraniol, piperitone, and citronellal are common terpenoid molecules found in EOs used to improve the health of animals. Their antimicrobial activity is modified by variations in phenylpropanoids, which are molecules synthesized from the amino acids’ tyrosine and phenylalanine. Phenylpropanoids are acids found in EOs used for animal health, such as vanillin, cinnamic, cinnamic, aldehyde, eugenol, chavicol, estragole, and safrole [36]. Various EOs have been shown to be effective antimicrobial agents against Gram-positive and Gram-negative bacteria, as well as yeasts and fungi, as can be seen in Table 1 [37,38].

The antimicrobial activity of EOs is associated with phenolic compounds due to the antimicrobial effect on altering the integrity permeability of the bacterial cell membrane [39]. Carvacrol and thymol are the primary components of EOs because they act against microorganisms by dispersing membrane-associated polypeptides through a lipophilic action on the cell membrane. Consequently, using EOs as dietary supplements assists in maintaining the health of horses and preventing digestive diseases [40].

### 3.1. Classification of EOs

The classification of EOs is determined by the chemical composition and consistency of their constituents. Based on these characteristics, they are categorized as balms, fluid essences, and oleoresins [41]. Balms have a thicker consistency, lack polymerization reactions, and are therefore somewhat volatile. Fluid essences are volatile liquids that are at room temperature. Oleoresin essences, which are liquid and quite viscous or semi-solid [41], are responsible for the concentrated aroma of plants. In addition, EOs are classified as artificial, natural, or synthetic based on their origin. In the case of artificial origin, the same essence is achieved through enrichment processes. There may be one or more components. Those obtained directly from the plant undergo no structural alteration (chemical or physical) [22,41]. Finally, synthetic EOs are created by combining various components produced by chemical synthesis processes [22,42].

The oil compounds contain an abundance of monoterpenes. Sesquiterpenoids are those that are primarily composed of sesquiterpenes, while phenylpropanoids are those that are rich in phenylpropanoids [42]. This classification is quite general in terms of EO classification. Nonetheless, it is useful for classifying and studying the photochemical properties of sesquiterpenes, monoterpenes, and phenylpropane. However, there are other classifications that are more specific and take into account specific chemical factors [43].

### 3.2. Mechanisms of Antimicrobial Action

Possible actions of EOs on a bacterial cell are primarily associated with membrane modifications, including electron transport, protein translocation phosphorylation, and ion gradient, among other enzyme-dependent reactions [44]. Although this process is not fully understood, the antimicrobial mechanisms performed by EOs in the rumen can dissolve the phospholipid bilayer of the cell membrane. The EOs can also reduce the ratio of acetate to propionate in the rumen, methanogenesis, and amino acid deamination [45]. Unlike dietary antibiotics, EOs do not alter the activity of the ruminal microbiota; consequently, their action mechanisms are more effective, and they do not lose their beneficial effect over time [46].

**Figure 3:**
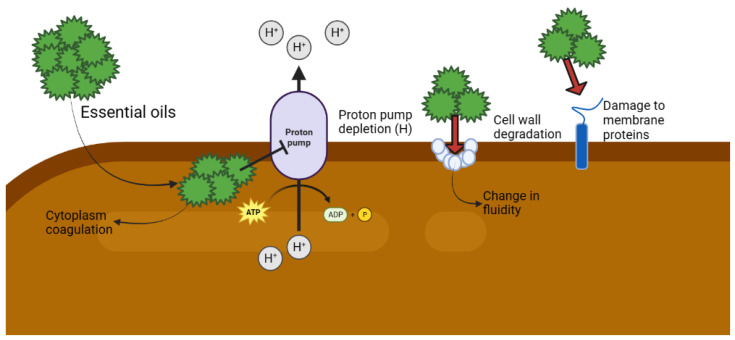
Possible antimicrobial action that occurs when using essential oils. ATP: adenosine triphosphate; ADP: adenosine triphosphate; P: Phosphate.

The antimicrobial activity of the chemical compounds present in EOs is not mediated by a single specific mechanism; among the various mechanisms of action, there is one that includes a methyl group, which drastically reduces the potential action of various compounds (Table 1). This is illustrated by the antimicrobial activity of the rumen when a diterpenoid antimicrobial agent interacts with water via its methyl group. In addition, the interaction between the EOs and the cell membranes alters the oil itself in a way that is more potent than the activity of its isolated components. However, it is also possible for the effects to be additive or antagonistic [16] (Figure 3).

Antioxidants are protective substances when the body has a low concentration of oxidizable substrates or reactive oxygen species, preventing or significantly delaying oxidative stress [19]. The presence of phenolic compounds in essential oils may be responsible for the antioxidant activity of these oils [36,47]. Anti-inflammatory actions and the inhibition of hydrophilic enzymes are characteristics of an antioxidant in the case of polyphenols with free radical stabilizing properties. The antioxidant activity of EOs may largely depend on their stability during storage, their rate of oxidation, and the concentrations of their more active components [48] (Table 1).

### 3.3. Microencapsulation

**Figure 4:**
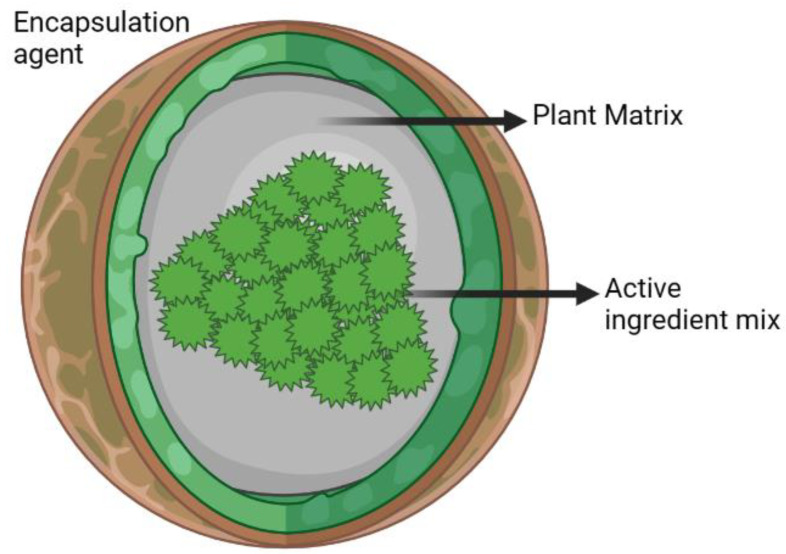
General diagram of microencapsulated capsule.

**Figure 5:**
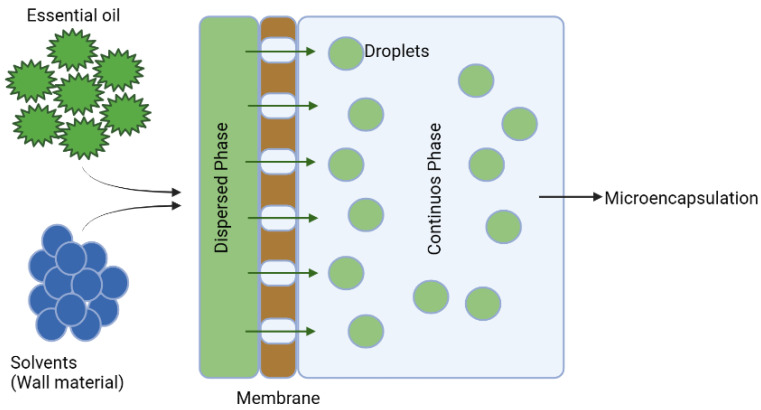
General scheme of membrane emulsification and encapsulation.

Microencapsulation is a technique for extending the stability of EOs during processing, manipulation, and storage [49] that involves coating small particles of liquid or gas, and sometimes solid, with a very thin layer called a wall (Figure 4). The encapsulation of food, vitamins, and EOs has increased because the encapsulated materials are protected from temperature and humidity, light, and oxidation, which could alter the composition of the active components. Microencapsulation can affect the quality of EOs due to its ability to permit the controlled release of active substances and the fact that packaging stabilizes pH and humidity, which reduces their volatile activity or vaporization significantly [49]. Microencapsulation can also prevent the solidification of EOs [41,50]. The technique utilized for microencapsulation is determined by the physical and chemical properties of the active compound. Galena gum and alginate are the most commonly used between the walls or matrices in microencapsulation. Galena gum is a vegetable gum in the form of a water-soluble polysaccharide; its molecule is linear and consists of glucose-based monomer bonds [50]. Alginates are anionic polysaccharides present in the cell walls of brown seaweed; their gel structure is used as a microencapsulation component [41,50] (Figure 5).

Maltodextrins, gum Arabic, gelatin, polyvinyl alcohol, and hydrophilic polymer are the primary compounds used in microencapsulation [51]; these compounds can be used as part of the microencapsulation wall. Other encapsulating materials include nylon membranes that primarily encapsulate enzymes. Another encapsulating material used in the food industry is quinosane. Alginate is a polymer derived from algae that is utilized for microencapsulation due to its high solubility and biodegradability. Alginate microparticles are prepared with distilled water and sodium alginate [50,51]. The encapsulation technique is dependent on the agent being encapsulated. In the case of lipids, lectins, waxes, monoglycerides, paraffin, hydrogenated oils, palm oil, soybeans, and cotton serve as effective encapsulants [40,50]. In the case of carbohydrates, the spray drying technique is used to encapsulate food ingredients in these substances. In the case of proteins, hydrocolloid foods such as sodium caseinates, soy protein isolates, and protein whey are commonly used for microencapsulation [50] (Table 1).

### 3.4. Encapsulation Techniques

**Figure 6:**
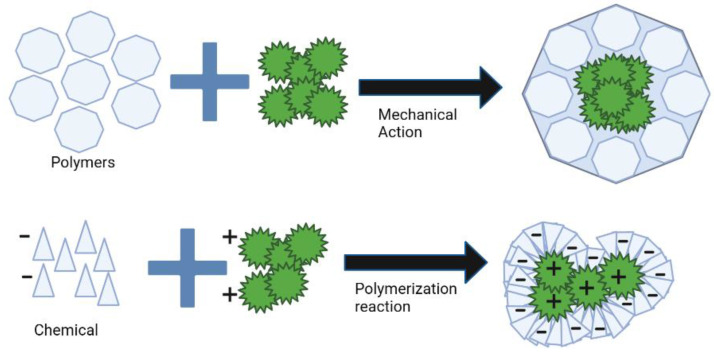
Chemical and physical methods for microencapsulation.

Encapsulation techniques are utilized in order to perform encapsulation in the most effective manner. These techniques consist of various approaches to the production of microcapsules. The development of science has essentially separated them into physical and chemical techniques. These techniques are distinguished by their advantages and disadvantages [52] (Figure 6). First, physical methods are characterized by the absence of polymerization reactions, as the materials are typically polymers; therefore, only the formation of microcapsules is performed mechanically. Secondly, chemical methods are characterized by the production of microcapsules via polymerization reactions of precursor materials such as pre-polymers, monomers, or materials involving chemical interactions [52] (Table 2).

Microencapsulates provide a method of controlled release, which necessitates the use of capsule-releasing components. When preparing microencapsulated compounds, it is necessary to take into account the microencapsulate’s selection, morphology, transition temperature, crossing, and swelling degree. The release of the capsules involves the release of the active substance bound to the capsule’s surface and the release of the active substance upon disintegration of the polymer matrix. When the matrix loses its integrity and the polymer chains reach a small enough size to be solubilized [18,53], dissolution occurs (Figure 5). The microencapsulates of EOs have the following beneficial impacts on horses’ digestion and health: (i) they make it easier to feed and reduce the unpalatable ingestion of feed due to the free EOs’ aroma; (ii) can be considered as one of the best alternatives to provide extra energy and improve the digestion of feed; (iii) maintain microbiota homeostasis and avoid metabolic and gastrointestinal disorders due to their ability to act against bacterial strains found in the equine hindgut; (iv) are easy to use and non-toxic to horses and affect microorganisms in the cecum and colon.

**Table 2:** Encapsulation techniques, physical, and chemical mechanisms.

Technique	Type	Process	Reference
Lyophilization	Physical	Lyophilization consists of two basic steps: freezing and drying. During the drying process, the water is removed from the sample. Loss of essential oils may be experienced in the drying process, due to temperature and the volatility of the essential oils.	[54]
Extrusion	Physical	The process requires the essential oil to flow under different conditions (depending on the technique) through a certain orifice. Extrusion techniques can be divided into the following: Hot-melt extrusion; Melt injection; Centrifugal/co-extrusion; Electrostatic/electrospinning; Particle from gas saturated solution (PGSS).	[52,55]
Fluidization	Physical	Fluidization is a method that keeps solid particles floating in a flow of gas. So, in this process, the particles are encapsulated using hot air in a coating chamber.	[56]
Spray dryer	Physical	It consists of atomizing essential oils with hot air, creating small particles, and evaporating the water. In this process, these small particles can also be covered with a “wall material”, such as polysaccharides.	[52]
Solvent removal	Physical	It consists of 4 steps: Dissolution of the compound and wall material into a suitable solvent; Emulsification of the solution; Solvent evaporation, creating solid particles; Recovery and drying of microspheres.	[52,57]
Coacervation	Chemical	This process is a coacervation that occurs between oppositely charged molecules. After polymerization, there are 2 separate liquid phases, a polymer-rich phase and polymer-depleted phase. Then, the polymer-rich phase is extracted. The process is strongly recommended for encapsulating essential oils.	[52,58]
Mini-emulsion polymerization	Chemical	In this process, monomer droplets are formed. These droplets act to promote the polymerization reaction, which results in the formation of polymeric particles.	[52,59]
Ionic gelation	Chemical	This technique is based on the ionic crosslinking of a polymer. When the essential oil is added to the reaction, it can be trapped inside the polymer.	[60]
Emulsification	Chemical	This process occurs by mixing two immiscible liquids. To form the encapsulation, some of the 2 liquids must be dispersed as droplets in the other.	[61]
Co-extrusion	Chemical	This process begins with the formation of droplets through vibration. These droplets then fall onto a solution with the gelling agent, resulting in the encapsulation of the active ingredient. Co-extrusion/gelation is widely used in the encapsulation of volatile substances.	[61,62]

## 4. Influence on Horses’ Cecal Fermentation

Due to the fact that horses are non-ruminant herbivores adapted to consuming plant fiber, their digestive processes are predominantly enzymatic, with ingesta fermentation occurring in the large intestine, cecum, and colon [53]. Horses have several feedings throughout the day, each of which consists of a relatively small amount of feed [18]. The large intestine is the organ that is responsible for the majority of the digestion, absorption, and fermentation of food [53,54]. The large intestine acts as a chamber to carry out the fermentation process, where microorganisms produce enzymes that are responsible for the hydrolysis of the feed; however, at the end of this process, not all of the synthesized or degraded nutrients are obtained [63] (Table 2).

Feed is broken down and converted into nutrients during digestion, thereby supplying the body with energy. When digestion is improper, nutrients from feed are not utilized, and the body produces toxins, resulting in gastrointestinal problems such as diarrhea, fatigue, and vitamin deficiency [64]. Therefore, EOs can reduce feed consumption when forages produce toxins and are unpleasant to the horses. For example, foals are the most susceptible to dietary changes; consequently, they must gradually ingest these substances to adapt [63].

Horses have enzymatic digestion, which occurs in the anterior intestine prior to the cecum; therefore, fermentation by the microbiota occurs in the cecum and colon, producing volatile fatty acids that are subsequently absorbed. This enzymatic and microbial digestion allows the horse to digest feed efficiently. The rate at which ingested feed passes through the stomach of a horse depends on the method of feeding. When horses consume a substantial meal, the time could be reduced to as little as 15 min. However, if fasting, stomach emptying will take 24 h. The mature equine’s intestine accounts for more than 60% of the total volume of the digestive tract, making it not only a critical water reservoir, but also important for microbial digestion of feed. The microbial populations in equine hindgut and cecum are almost similar to those found in the rumen, and the common bacterial species are cellulolytic (*Fibrobacter succinogenes*), hemicellulolytic (*Butyrivibrio fibrisolvens*), proteolytic (*Ruminobacter amilophilus*), methane-producing species (*Methanobacterium formicicum*), amylolytic species (*Streptococcus bovis*), ammonia-producing species (*Selenomonas ruminantim*), pectinolytic species (*Prevotella ruminicola*), lipid users (*Treponema bryantii*), sugar users (*Lactobacillus vitulinus*), acid users (*Megasphaera elsdenii*), and ureolytic species (*Butyrivibrio* sp.) [65,66]. Growing horses require a low-fiber, easy-to-digest diet because, unlike adult horses, their cecal digestion is less developed and their microbes are still developing [40] (Figure 6).

## 5. Influence on Cecal Microbial Population

The intestinal microbiota has a significant effect on animal health due to its susceptibility to external factors, which can cause diseases or digestive imbalance. The horse intestine is crucial for microbial fermentation, digestion, and absorption of nutrients, because these processes depend on pre-cecal digestibility, which influences the rate of ingesta passage; the retention times of pre-cecal digestion in the intestine influence digestion due to microbial activity and water absorption. In contrast to draft breeds, the retention time (MRT) for light horse breeds is less than 26–10 h [67]. The influence and interaction of the microbial population in the large intestine are crucial. The pre-cecal digestibility is dependent on the post-gastric placement of fermentative activity, substrate availability, microbial growth, and degradation [16]. Approximately 30% of the microbial population in the colon and cecum are anaerobes. These bacteria can generally be categorized as cellulolytic, proteolytic, or methanogenic [16,68].

Horses are herbivores with a fermenting hindgut (cecum–colon) that obtain nutrients from a high-fiber diet. Most microorganisms in the cecum–colon are strict anaerobes, including fungi, bacteria, and protozoa. Diet influences the composition, number, and structure of the microbe species inhabiting the cecum–colon [68]. The bacteria discovered in the cecum–colon can be categorized based on the substrate they utilized or their nutritional needs. They can be categorized according to whether they degrade cellulose, starch, hemicellulose, sugars, proteins, intermediate acids, pectins, or lipids [15].

## 6. Influence on Methane Production

Methane (CH_4_) is a gas that is classified as a greenhouse gas. The CH_4_ consists of two or more atoms that are joined but have enough space to “vibrate” when they absorb heat, so that when they begin vibrating, they emit radiation that is subsequently absorbed. It causes a greenhouse effect by producing and retaining heat close to the Earth’s surface [60,61]. Whether from natural or anthropogenic sources, greenhouse gas emissions are released into the atmosphere. Therefore, reducing emissions of these gases can aid in the prevention of global warming [69,70] and the development of a more sustainable system for horse production. Fermentation in the hindgut (cecum–colon) produces CH_4_ [71,72], which significantly contributes to global warming [16].

In recent years, the concentration of CH_4_ in the atmosphere has more than doubled; the income index can be used to determine these concentrations [62]. It is important to note that CH_4_ is non-toxic, it is highly flammable and reacts with oxidizing agents, halogens, and some halogenated compounds, which pose the greatest threat. Human, agricultural, and animal activities account for approximately sixty percent of the world’s CH_4_ emissions [61]. Methanogenesis is an anaerobic organism’s metabolic pathway (methanogenic organisms include *Methanobacterium formicicum* and *Methanomicrobium mobile*). CH_4_ is formed from CO_2_ and H_2_O production during digesta fermentation by cecal microorganisms. Animal digestion and defecation processes (cecum–colon fermenters) account for 17% of this gas [61,62]. Rice fields are another significant contributor to the CH_4_ content of the atmosphere, and recent estimates put the global emissions of this gas at 36 Tg/year [73].

The EOs have an effect on the equine metabolism; some can act as antioxidants, while others can stimulate digestion or increase the regulation of gastrointestinal metabolism, and others can improve nutrient absorption. Nevertheless, by acting synergistically in the metabolism, EOs can facilitate the absorption of nutrients by altering the fermentation process. As a cecum–colon fermenter, the decrease in CH_4_ production in horses will never be as pronounced as in ruminants. Nonetheless, this fermentation process may aid in reducing enteric CH_4_ production in horses [16]. Few studies have examined the effects of EOs on horses. Similar to those found in the rumen, the equine large intestine microbiome [74] consists of protozoa, bacteria, fungi, archaea, and bacteriophages [75]. Thus, EOs have the potential to manipulate the cecum–colon microbial population and fermentation pattern [75,76]. The EOs reduce amino acid deamination, the ratio of rumen acetate to propionate, and methanogenesis [45].

## 7. Influence on Horse Nutrition

The horse derives its energy from ATP (adenosine triphosphate) from the fiber-rich diet it consumes, and the horse’s digestive system includes various processes and organs that are responsible for degradation. After chewing, the feed and saliva are transported through the esophagus to the stomach. The horse’s stomach is small, but it is where feed is stored and partially digested; it is important to note that most nutrients, including proteins and carbohydrates, are not absorbed in the stomach—most are only partially digested. The feed is transported from the stomach to the small intestine, where it acts as a conduit to move a large number of carbohydrates that have not yet been digested to pass first to the cecum, where fermentation occurs, and then to the large intestine, where hydrolysis of nutrients occurs. Cecum and colon constitute the largest and most intricate portion of the digestive tract. The large intestine serves various functions, including as a water reservoir [18,50].

Feeding horses is essential in order to meet their daily nutritional needs, which include proteins, carbohydrates, vitamins, minerals, and water. Indeed, these vary according to age, size, and work performed. High-intensity activities necessitate a greater energy supply for horses. The forage-based diet that they normally consume does not meet the additional requirements for vegetable oils, which not only increase the proportion of extra energy, but also provide health benefits that improve feed digestion [41].

Adequate nutrition is a fundamental part of the health of equines that have daily agricultural work or that have undergone abrupt diet changes and diets with concentrated feed. Therefore, maintaining microbiota homeostasis is important to avoid metabolic and gastrointestinal disorders. There are EOs with the ability to act against bacterial strains found in the equine hindgut that could have important potential in maintaining homeostasis and, therefore, animal health [31,33,34] (Table 1).

EOs are user-friendly, made entirely of natural substances with high concentrations of active ingredients, and may have pleasant odors for equines. However, EOs’ aroma may reduce the feed palatability and thus affect feed intake. Because horses have a keen sense of smell and quickly reject unpalatable feed [41], the pH of the EOs that are administered to them must be taken into account (Table 3).

## 8. Conclusions

The susceptibility of horses to changes in diet, essential oils could be used to meet their energy needs and prevent disease. Because they are easily absorbed and can be applied topically, EOs provide numerous health benefits for horses through body regulation, cell regeneration, and immune system support. These substances have antibacterial, anti-inflammatory, and analgesic properties, in addition to improving the absorption of nutrients in horses. It is important to note that EOs have limitations due to their high volatility and tendency to degrade. This is where microencapsulation becomes a method to avoid these drawbacks. Microencapsulation can be used to encapsulate solid, liquid, and gaseous substances. In addition, its content is released in a controlled manner under specific conditions, and it can also protect the products it contains from light and oxygen. Horses have a keen sense of smell and are quick to reject unpalatable feed, so microencapsulation could help make it easier to incorporate EOs into the diet. For this reason, the microencapsulation of EOs is one of the best alternatives to provide extra energy and improve the digestion of feed. EO microcapsules can maintain microbiota homeostasis and avoid metabolic and gastrointestinal disorders due to their ability to act against bacterial strains found in the equine hindgut. EO microcapsules are easy to use and non-toxic to horses, affecting microorganisms in the cecum and colon. Essential oils reduce amino acid deamination, ruminal acetate to propionate ratio, and methanogenesis. 

## Figures and Tables

**Table 1:** Different biological effects of essential oils.

EOs	Effect	Main Composition %	Reference
*Jungia paniculata*	High MICs, inhibition of parasite growth (*L. amazonensis*)	β-Caryophyllene (35.91%), Caryophyllene oxide (36.49%), α-curcumene (3.85%)	[22]
Tangerine (Dancy variety and commercial Gerch)	Bactericidal of *Staphylococcus aureus*, *Listeria monocytogenes* and *Bacillus subtilis*	EOs pure Tangerine (100%)/linalool (dimethyl-2,7-octadien-6-ol.) and thymol (2-isopropyl-5-methylphenol) chemotypes a	[23,24]
Tangerine (Dancy variety and commercial Gerch)	MICs for *Bacillus subtilis* 9% and 19%, respectively; for *Staphylococcus aureus* and *Listeria monocytogenes* 7%	EOs pure Tangerine (100%)/linalool (dimethyl-2,7-octadien-6-ol.) and thymol (2-isopropyl-5-methylphenol) chemotypes a	[23,24]
*Eucalyptus* spp. (*Eucalyptus*)	*Staphylococcus aureus* CMI 6.8 µL/ML MBC (6.8 µL/mL), *Escherichia coli* CMI (13.2 µL/mL), MBC (13.2 µL/mL)	Eucalyptol (57.85%), α-pinene (22.81%), α-terpinyl acetate (3.72%), β-Myrcene (1.85%), Viridiflorol (1.6%), β-pinene (1.53), Aromadendrene (1.49%), α-Terpineol (1.27%)	[24]
*Citrus lemon* (L.) Osbeck (Lemon)	*Staphylococcus aureus* MICs 7.6 µL/Ml MBC (7.6 µL/mL) *Escherichia coli* MICs (14 µL/mL), MBC (13.2 µL/mL)	Limonene (58.17%), β-pinene (13.22%), γ-Terpinene (11.72%), β-Myrcene (1.75%), Octanal (1.67%), Citronellal (1.5%), α-Terpineol (1.19%)	[24]
*Origanum vulgare* (Labiate) (oregano)	Antimicrobial effect on Gram-positive bacteria *Staphylococcus aureus* and *Bacillus cereus* and on Gram-negative bacteria	9,12-octadecadienoic acid (8.29%),9, 12, 15. octadecatrienal (8.29%), Cis sabinene hydrate (18.66%), 4-terpineol (9.43%), Carvacrol (7.72%)	[25]
*Piper hispidum* (matico hoja lisa)	Antibacterial activity in *X. albilineans*	a-Phellandrene (22.30%) a-Pinene (14.82%) Eucalyptol (15.49%) NI (CHO) (12.90%)	[22,26]
*Pimpinella anisum* L.	Antibacterial activity on *Bacillus subtilis*, *Pseudomona aeruginosa*, *Staphylococcus aureus*, *Streptococcus pyogenes*, *Escherichia coli*, and *Klebsiella pneumoniae*	canfeno (0.11%), linalol (0.11%), 4-ciclopropil-2-metoxifenol (0.19%) metil chavicol (97.76%), B-cariofileno (0.12%), germacreno-D (0.44%)	[27]
*Thymus vulgaris* (thyme)	70% inhibition against *S. aureus* strain and 20% *E. faecalis* strain	β-pineno (29.0%), 1,8-cineol (21.5%), and o-cimeno (17.9%)	[28]
*Curcuma longa* (cúrcuma)	70% inhibition against *S. aureus* strain and 50% *E. faecalis* strain	turmerona (36.9%), α-turmerona (18.9%), and β-turmerona (13.6%);	[28]
*Eucalyptus globulus*	Inhibition of salmonella, *Bacillus subtilis*, *Enterococcus faecalis*, *Escherichia coli*	1,8-Cineol or Eucalyptol (82.27%), Limonene (3.70%), α-Pinene (3.16%)	[29]
*E. camaldulensis*	Higher concentrations of the oil inhibit bacterial strains of *Bacillus subtilis*, *Enterococcus faecalis*, *Escherichia coli*	1,8-Cineol (77.41%), Terpinen-4-ol (3.68%), α-Pinene (3.64%), Limonene (3.21%)	[29]
*Myrcianthes leucoxyla*	Antioxidant activity from 500 ppm when inhibition percentages higher than 60% are reached	Pineno (28.40%), 1,8-Cineol (15.70%), Z-Cariofileno (3.79%), Cariofileno Guaiol (3.13%)	[30]
*Lippia graveolens*	Antimicrobial activity, antifungal, antibacterial, antioxidant, antiprotozoal	1,8-Cineol, phellandrenes, p-cymene, terpinenes, carvacrol, thymol and their ethers and esters, b-caryophyllene	[31]
*Cinnamomum zeylanicum* (Cinnamon)	Inhibition to salmonella strains, mainly sensitive to concentrations of cinnamon essential oil at concentrations of 50% or higher	eugenol, present in 70–95%	[32]
*Chenopodium ambrosioides* (Epazote)	Analgesic effects and against arthritis	1-methyl-4-isopropyl-2,3-dioxa bicyclo[2.2.2]hept-5-ene) occurs in 60–80% of the essential oil of *C. ambrosioides*, and in 1% by fresh weight	[33]
*Calycolpus moritzianus*	Increased antioxidant activity	monoterpenes α-Pinene, Eucalyptol and α-Terpineol	[34]
*Minthostachys mollis*	Presents antioxidant activity, but in concentrations higher than 200 ppm	1,8-Cineol or Eucalyptol (6.39%)	[34]
*Bursera graveolens* Triana	Acetylcholinesterase inhibitory activity	Major compound (viridiflorol)	[35]

Essential oils and biological effects. EOs: essential oils; MICs: minimum inhibitory concentrations; MBC: minimum bactericidal concentration).

**Table 3:** Essential oils used in horses’ diets and dosages customarily used.

Type of Essential Oil	Dose	Effect	Reference
Roman chamomile Marjoram	Apply 20–30 drops of pure oil (dilute with V-6 vehicular oil if phenol-rich oils are used).	Can be placed in a capsule and mixed with food. It helps to combat nerve problems and anxiety in horses.	[65]
Mint Cypress	Essential oil drops on the coat approximately 10 cm in size.	Therapeutic application for horses.	[66]
Valeriana	20–30 pure drops per application.	Relieves anxiety in animals; the calming effect lasts from days to weeks.	[77]
Marjoram	Application of essential oil drops on the coat, approximately 10 cm in size.	Therapeutic application for horses.	[65]
Gauteria	Apply diluted in a vegetable oil such as almond oil or even arnica, which is also an anti-inflammatory.	It is a powerful anti-inflammatory and analgesic, but contraindicated during pregnancy and lactation.	[66]
Rosemary chemotype camphor	Apply diluted in a vegetable oil such as almond oil or even arnica, which is also anti-inflammatory.	Muscle relaxant and analgesic, ideal for muscle contractures, torticollis, and other types of pain.	[66]
BIOBRON is a food supplement based on a synergistic blend of essential oils.	20 mL/day per 100 kg of live weight for 5 days (there are also more concentrated versions).	It helps animals recover due to essential oils’ expectorant and fluidizing properties. Stimulates food consumption and general health thanks to plant extracts and vitamin C.	[65]
Soybean or sunflower oil	20 percent or 450 mL daily of the total diet.	Contributes to the proper growth of the foal. Enhances absorption of vitamins A, D, E, K, and linoleic acid. Prevents colic.	[19]
Corn oil	90 mL in a single meal; after 3 days, 90 mL in each meal and continue to increase gradually until reaching the desired level.	Maintains reserves of muscle glycogen and glycemia levels for a longer time.	[77]

## Data Availability

All other material is from published literature referenced in the reference list.
